# miR-19a: An Effective Regulator of SOCS3 and Enhancer of JAK-STAT Signalling

**DOI:** 10.1371/journal.pone.0069090

**Published:** 2013-07-22

**Authors:** Aideen S. Collins, Claire E. McCoy, Andrew T. Lloyd, Cliona O’Farrelly, Nigel J. Stevenson

**Affiliations:** 1 School of Biochemistry and Immunology, Trinity College, University of Dublin, Dublin, Ireland; 2 School of Medicine, Trinity College, University of Dublin, Dublin, Ireland; University of Hong Kong, Hong Kong

## Abstract

Suppressors of cytokine signalling (SOCS) proteins are classic inhibitors of the Janus kinase-signal transducer and activator of transcription (JAK-STAT) pathway. Many cytokines and pathogenic mediators induce expression of SOCS, which act in a negative feedback loop to inhibit further signal transduction. SOCS mRNA expression is regulated by DNA binding of STAT proteins, however, their post-transcriptional regulation is poorly understood. microRNAs (miRNAs) are small non-coding RNAs that bind to complementary sequences on target mRNAs, often silencing gene expression. miR-19a has been shown to regulate SOCS1 expression during mutiple myeloma and be induced by the anti-viral cytokine interferon-(IFN)-α, suggesting a role in the regulation of the JAK-STAT pathway. This study aimed to identify targets of miR-19a in the JAK-STAT pathway and elucidate the functional consequences. Bioinformatic analysis identified highly conserved 3’UTR miR-19a target sequences in several JAK-STAT associated genes, including SOCS1, SOCS3, SOCS5 and Cullin (Cul) 5. Functional studies revealed that miR-19a significantly decreased SOCS3 mRNA and protein, while a miR-19a antagomir specifically reversed its inhibitory effect. Furthermore, miR-19a-mediated reduction of SOCS3 enhanced IFN-α and interleukin (IL)-6 signal transduction through STAT3. These results reveal a novel mechanism by which miR-19a may augment JAK-STAT signal transduction via control of SOCS3 expression and are fundamental to the understanding of inflammatory regulation.

## Introduction

The JAK-STAT pathway mediates important biological mechanisms, including inflammation, cell proliferation and anti-viral activity, and is stimulated by receptor binding of cytokines, such as IFNs and IL-6 [1,2]. Activation of JAKs (JAK1-3, tyrosine kinase 2) leads to STAT phosphorylation, dimerisation and translocation to the nucleus, where they bind responsive DNA elements, often inducing mediators such as pro-inflammatory cytokines and IFN stimulated genes (ISGs) [3,4]. The JAK-STAT pathway is under tight regulation by the induction of SOCS proteins. SOCS proteins silence the pathway by acting as pseudo-substrates that block JAK kinase ability, binding to the receptor to prevent STAT interaction and targeting proteins for proteasomal degradation [5].

SOCS form Elongin C-Cullin–SOCS box (ECS)-type complexes that function as E3 ubiquitin ligases and target specific proteins for ubiquitin-mediated degradation. This is achieved when Elongin B binds Elongin C, which bridges the substrate recognised by the SOCS protein to a Cul scaffold protein [6]. SOCS3 targets receptors for proteasomal degradation following association with Cul5 and Elongin BC [7].

SOCS3 has been reported to regulate many signalling pathways, including those activated by IL-6 and IFN-α [8,9]. More recently, we have shown that SOCS3 also inhibits granulocyte macrophage-colony stimulating factor (GM-CSF) and IL-4 signalling to regulate dendritic cell (DC) maturation [10] and that SOCS3 targets focal adhesion kinase (FAK) and Ras homolog gene family, member A (RhoA) to block migration towards the allergic chemokine CCL11 [11]. A key role for SOCS3 in the regulation of IL-6 signalling was identified by conditional knock out (KO) of SOCS3 in murine liver and macrophages, resulting in prolonged activation of STAT1 and STAT3 [8], while an inhibitory role for SOCS3 in IFN signalling has been widely documented to confer resistance to treatment for Hepatitic C virus (HCV) infection [12]. Dysregulation of the JAK-STAT pathway has been implicated in haematopoietic, autoimmune and inflammatory disorders and cancer, further highlighting the necessary and vital role of SOCS proteins in regulating the initiation, duration and magnitude of cytokine signalling [13]. SOCS3 KO mice die embryonically as a result of placental defects, demonstrating an essential role for SOCS3 in controlling cell homeostasis [14]. Even though SOCS3 is an established regulator of the JAK-STAT pathway, its own regulation is poorly understood.

miRNAs are small non-coding RNAs that post-transcriptionally regulate expression of ≥30% of protein-coding genes [15]. miRNAs regulate intracellular pathways of numerous inflammatory mediators [16] and their role in the regulation of immune responses has been widely documented, including the presence of miR-181a in the thymus and miR-223 in the bone marrow being implicated in the differentiation of pluripotent hematopoietic stem cells [17,18]. Additionally, miR-155 has been shown to be induced by viral and bacterial pathogens, as well as inflammatory cytokines, suggesting it to be a component of the innate immune response [19,20]. Even though the function of these novel regulators in several processes is now well described, their effect on the JAK-STAT pathway has not been explored in any great detail. miR-155 has been shown to target SOCS1 in macrophages, positively regulating the host anti-viral response by promoting type I IFN signalling [21]; and in breast cancer cells, miR-155 suppression of SOCS1 plays an oncogenic role resulting in the constitutive activation of the JAK-STAT pathway [22]. Furthermore, miR-155-mediated SOCS1 suppression has been shown to enhance JAK-STAT signalling required for Treg/Th17 cell differentiation [23], and is linked to increased IL-12 production in DCs [24]. LPS-induced miR-155 reduced SOCS1 expression in microgial cells, resulting in enhanced inflammatory responses, such as cytokine and nitric oxide production [25]. miR-9 has been shown to target SOCS5 [26], while miR-98 targets SOCS4 [27], and *Porphyromonas gingivalis*-induced miR-203 targets SOCS3 [28]. Interestingly, miR-19a is induced by the anti-viral JAK-STAT signalling cytokine, IFN-α [29] and reduces SOCS1 expression, which may be linked to enhanced IL-6 signalling in multiple myeloma [30], suggesting a significant role for miR-19a in the regulation of the JAK-STAT pathway. Since the function of miR-19a was largely unknown, we further investigated its role in the regulation of the JAK-STAT pathway. Our study reveals that miR-19a targets SOCS3, a strong regulator of the JAK-STAT pathway, as demonstrated by a dramatic reduction of SOCS3 mRNA and loss of protein expression, together with increased activation of the SOCS3 target, STAT3.

## Materials and Methods

### Identification of miR-19a binding sites in components of the JAK-STAT signalling pathway

The Kyoto encyclopedia of genes and genomes (KEGG) database was used to identify proteins involved in JAK-STAT signalling. InnateDB was used to identify genes that interact with SOCS proteins. TargetScan (Version 5) was used to identify the proteins containing a target sequence for miRNAs which belong to the miR-17-92 cluster (miR-17, miR-18a, miR-19a, miR-20a, miR-19b-1, and miR-92-1), in their 3’UTR. TargetScan predicted biological targets of miRNA by searching for the presence of conserved 8 and 7 base sites that match its seed region.

### Transfection

The adherent hepatocyte cell line, Huh7, was chosen for its responsiveness to both IFN-α [31] and IL-6 [32], its transfectable nature and the fact that Huh-7 cells express low endogenous levels of miR-19a, thereby giving us an appropriate tool to investigate the role of double-stranded miR-19a mimic. Huh7 cells were grown in Dulbecco’s Modified Eagle Media (DMEM), supplemented with 10% Fetal Calf Serum (FCS), 2mM L-glutamine, 250U/ml penicillin and 250µg/ml streptomycin. Cells were grown in 10% DMEM containing no antibiotics at a density of 2 x 10^5^ in a 6-well plate, prior to transfection. Huh7 cells were transfected with 5nM of double-stranded miR-19a or miR-17 mimics and microRNA mimic negative control 1 (MMNC) (Ambion) for 24, 48 and 72h using Lipofectamine 2000 (Invitrogen), according to the manufacturer instructions. MMNCs are random, scrambled sequence double-stranded molecules that have been extensively tested in human cell lines and tissues and validated to not produce identifiable effects on known miRNA function. The double-stranded miR-19a mimic is a synthetic microRNA molecule designed to mimic endogenous mature miR-19a. Prior to IFN-α stimulation, cells were rested for 2h in DMEM supplemented with 2% FCS. Cells were then stimulated with 1000U/ml of IFN-α (Roche) or 10ng/ml of IL-6 (R&D Systems). Experiments were carried out three times separately.

### Quantitative real-time PCR (qRT-PCR)

Total RNA was isolated from Huh7 cells following the Trizol manufacturer’s protocol (Invitrogen). 1µg of total RNA was reverse transcribed into cDNA using an OmniScript^TM^ III first strand synthesis kit with oligo-dT primers according to the manufacturer’s instructions (Invitrogen). A total of 40ng of cDNA was subsequently used for each qRT-PCR reaction. PCR amplification was performed using specific primer pairs (Figure **S1**). qRT-PCR was performed using the Sybr Green based fluorescent method and the MX3000P® qRT-PCR system (Stratagene Corp) using cycling parameters: 95°C for 30s, 60°C for 1min and 72°C for 30s for 40 cycles, followed by amplicon dissociation. Gene amplification was normalised to Ribosomal protein 15 (RPS15) which was selected as the most stably expressed gene across a panel of potential house keeping genes. Data analysis was carried out using the 2^-ΔΔCT^ method. Standard deviation (S.D.) of the relative gene expression was compared to the MMNC. *P* values were calculated using paired t-tests.

300ng of RNA was reverse transcribed using specific Applied Biosystems TaqMan micro-RNA stem-loop primers for miR-19a, to generate cDNA. Applied Biosystems TaqMan miRNA specific probes were then used to quantify the levels of miR-19a in each sample, according to the manufacturer’s instructions. RNU6B (Applied Biosystems) was selected as the endogenous control. miRNA expression was measured on the 7900HT FAST RT-PCR system. Data analysis was carried out using the 2^-ΔΔCT^ method. Data analysis was carried out as described above.

### Immunoblotting

Following transfection of double-stranded miR-19a mimic (pre-miR-19a) and MMNC, and stimulation with IFN-α and IL-6, cells were harvested in radio-immunoprecipitation assay (RIPA) lysis buffer, supplemented with aprotinin (5µg/ml), leupeptin (5µg/ml), phenylmethanesulfonylfluoride (PMSF) (1mM) and Na _3_VO_4_ (1mM), on ice for 15min. Extracts were pelleted at 12,000xg at 4^o^C for 15min. A bicinchoninic acid (BCA) protein assay was carried out and protein concentration was calculated from a known BCA standard curve. All samples were normalised using the values generated from the standard curve relative to their protein concentrations using RIPA buffer. Total lysates were resolved by polyacrylamide gel electrophoresis (PAGE) and protein was transferred from the gel to a Polyvinylidene diflouride (PVDF) membrane using semi-dry transfer. The membrane was blocked for 1h with 5% powdered milk or 3% bovine serum albumin (BSA) in phosphate buffered saline (PBS) supplemented with 0.15% Tween-20 (PBST). Primary antibodies were diluted 1:1000 in 5% milk (SOCS3 and γ-tubulin) or 3% BSA (pSTAT3-Tyr705) and left incubating at 4^°^C overnight. The following morning blots were washed 3 times in PBST for 10min before being incubated at room temp for 1h, with relative secondary antibody, made to a concentration of 1:5000 with milk or BSA. Primary antibodies SOCS3 (Abcam), pSTAT3-Tyr705 (Cell Signaling) and γ-tubulin (Sigma) and infrared dye-labelled secondary antibodies, goat anti-rabbit and goat anti-mouse (Li-Cor), were used for detection. Proteins were analysed on an Odyssey Infrared Imaging system using near-infrared (NIR) fluorescence detection. Densitometry was calculated using Li-Cor software.

### Reporter assay

Human SOCS3 3’UTR (SOCS3_UTR) was amplified from human genomic DNA, (isolated from B cells), using forward and reverse primers, (designed from the human SOCS3 3’UTR sequence [Figure **S2**]), which included EcoRI restriction enzyme sites.

Primer sequences:

5’-CGGAATTCgggtaaagggcgcaaagg-3’ and 5’-CGGAATTCtaaattactgaaatgtttcttttctga-3’,

(lower case = SOCS3 3’UTR sequence, capitalised = EcoRI restriction site sequence.) 

The amplified product was cloned into the pRL R*enilla* luciferase reporter plasmid (Promega) using EcoR1 restriction digest. The mutant SOCS3 3’UTR (mSOCS3_UTR) was generated from this plasmid using forward and reverse primers designed from the SOCS3 3’UTR sequence (Figure **S2**): Primer sequences:

(5’-gtattctgtgtcaggtattgggctg-3’ and 5’-aaatataaatatgACGaaagtttgacttggattgg-3’,

(lower case = SOCS3 3’UTR sequence, capitalised = base changes within the miR-19a seed region.) 

SOCS3_UTR or mSOCS3_UTR (200ng) and firefly luciferase expression plasmid control (15ng) were reverse transfected into Huh7 cells seeded at 5 x 10^4^/ml in 6 well plates using Lipofectamine 2000 according to the manufacturer’s instructions. Three hours later, cells from each 6 well were re-plated into 12 wells of a 96 well plate and transfected in biological triplicate with double-stranded miR-19a mimic (pre-miR-19a) and/or MMNC control or miR19a antagomir (AMO 19) or antagomir control (AMO C) with indicated concentrations. 24h later, cells were lysed and analysed for *Renilla* luciferase expression. Data was normalised to firefly luciferase control and represented as fold induction over MMNC control or AMO C.

## Results

### Prediction of miR-19a targets in components of the JAK-STAT signalling pathway

**Figure 1:**
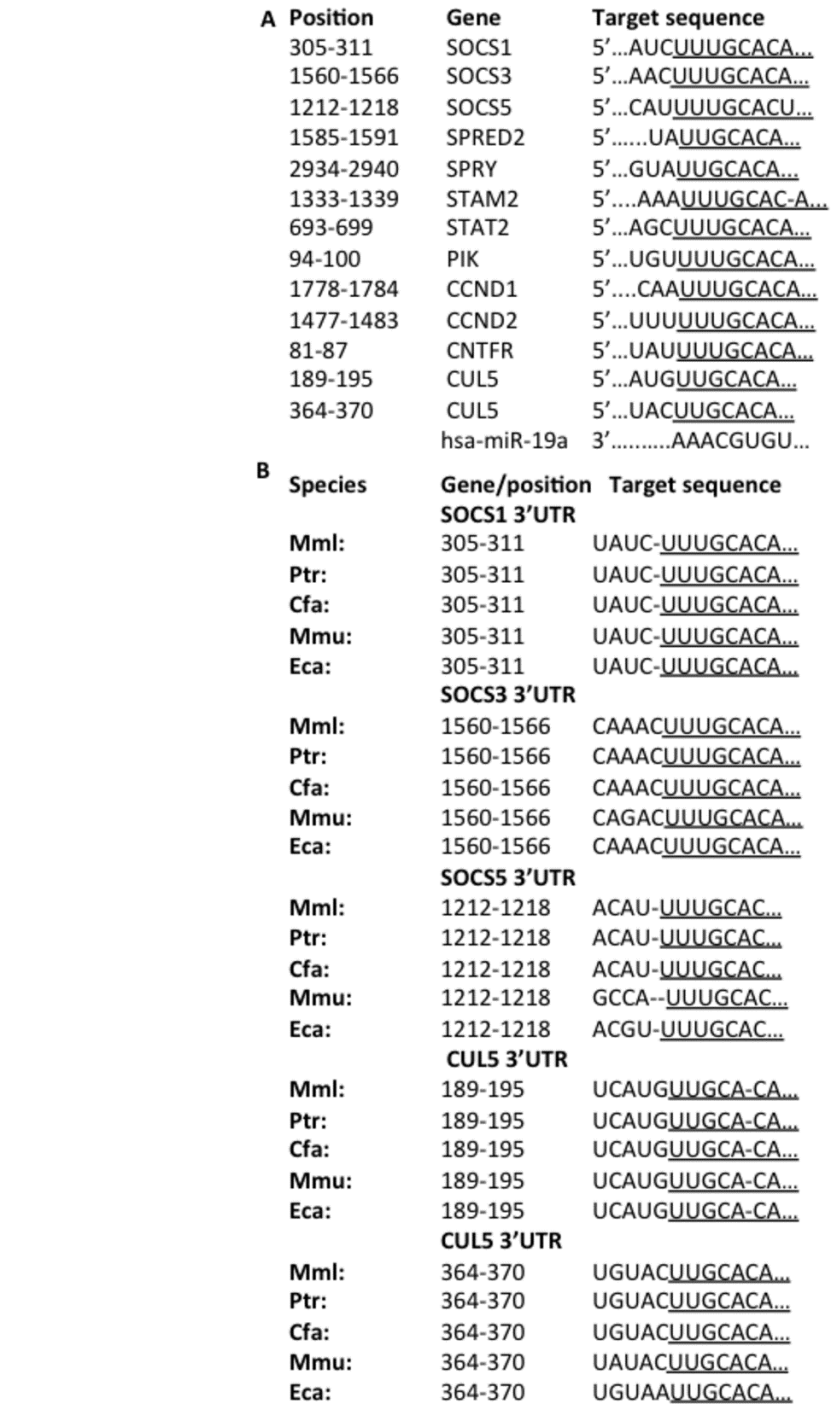
Components of the JAK-STAT pathway contain miR-19a target sites in their 3’UTRs. (A) Sequence alignment of miR-19a and its target sites in the 3’UTRs of 12 JAK-STAT-associated genes. (B) miR-19a binding sites in SOCS1, SOCS3, SOCS5 and Cul5 are conserved among rhesus (Mml), chimpanzee (Ptr), dog (Cfa), mouse (Mmu) and horse (Eca).

A combination of KEGG database (containing pathway maps representing molecular interaction and reaction networks) and InnateDB database (containing innate immunity interactions and pathways), identified 158 proteins in the JAK-STAT pathway (Figure **S3A**), while TargetScan (software for predicting microRNA targets) predicted 938 target genes for miR-19a. We identified 12 protein coding genes associated with the JAK-STAT pathway to contain miR-19a target sequences in their 3’UTR (Table 1). TargetScan revealed that all 12 genes, including the regulatory SOCS1, SOCS3, SOCS5 and Cul5, had the 7-8 seed sequence of miR-19a in their 3’UTRs (Figure 1A), but, interestingly only 2 of the 12 genes had target sequences in their 3'UTRs for other miRNAs within the miR-17-92 cluster (miR-17, miR-18a, miR-20a, miR-19b-1, and miR-92-1). Specifically, signal-transducing adaptor molecule (STAM) 2 has a predicted binding site for miR-18a, and cyclin (CCN) D1 has a predicted binding site for miR-17 and 20ab. While the miR-19a binding site was common across SOCS1, SOCS3 and SOCS5 3’UTRs, other miRNA binding sites were also identifed in their 3’UTRs (Figure **S3B**). Furthermore, we found that the entire 3’UTR of these genes (data not shown) and each of the these regulatory miR-19a binding site genes were conserved across several species, including chimpanzee (Ptr), rhesus (Mml), dog (Cfa), mouse (Mmu) and horse (Eca) (Figure 1B), strongly indicating a precise and important role for miR-19a in the regulation of JAK-STAT pathway components.

**Table 1:** JAK-STAT proteins containing miR-19a target sites.

**Gene**
SOCS1
SOCS3
SOCS5
SPRED2
SPRY3
STAM2
STAT2
PIK3R3
CCND1
CCND2
CNTFR
CUL5

Twelve genes, (suppressor of cytokine signalling [SOCS] 1, SOCS3, SOCS5, sprouty-related, EVH1 domain-containing protein [SPRED] 2, sprouty [SPRY] 3, signal-transducing adaptor molecule [STAM] 2, signal transducer and activator of transcription [STAT] 2, phosphatidylinositol 3-kinase regulatory subunit [PIK3R] 3, cyclin [CCN] D1, CCND2, ciliary neurotrophic factor receptor [CNTFR] and Cullin [CUL] 5), coding for protein components of JAK-STAT signalling pathway that contain miR-19a targets sites in their 3’ UTRs.

### Validation of miR-19a targets of the JAK-STAT pathway

**Figure 2:**
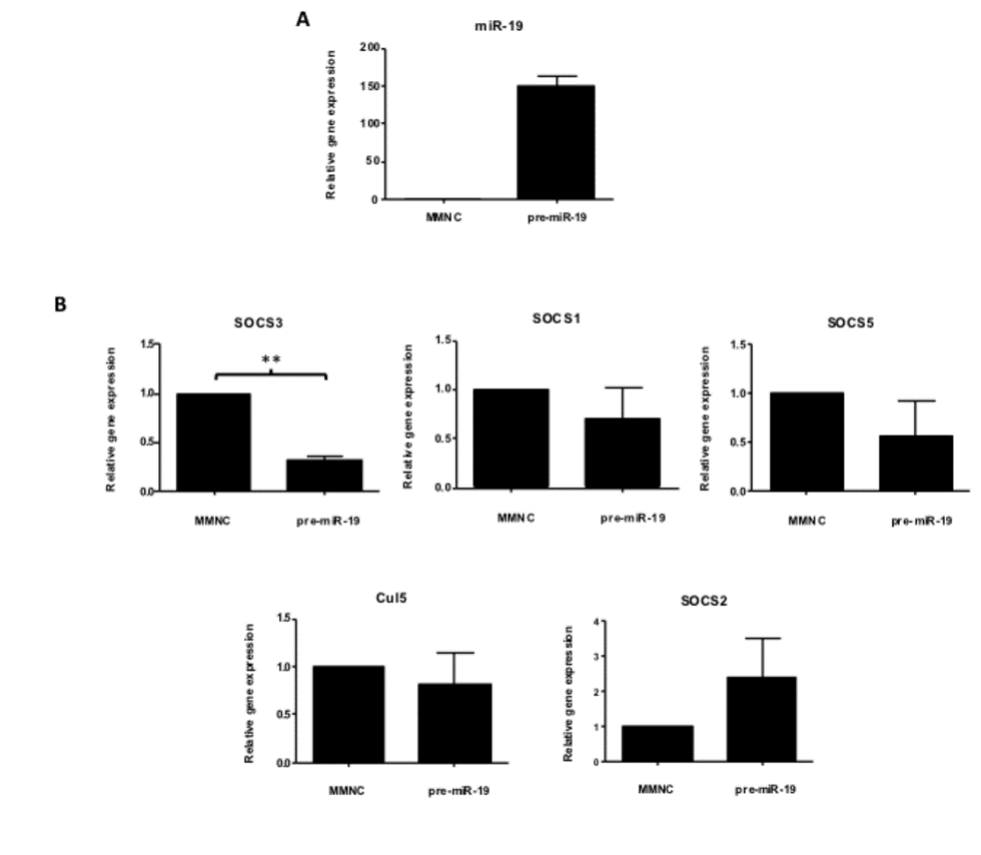
miR-19a silences SOCS3 of the JAK-STAT pathway. qRT-PCR analysis of (A) miR-19a (n=3) and (B) SOCS3, SOCS1, SOCS5, Cul5 and SOCS2 (n=3) mRNA in Huh7 cells transfected with double-stranded miR-19a mimic (pre-miR-19a) compared to cells transfected with MMNC. Mean ± S.D of the relative gene expression was compared to the MMNC, which was normalised to 1. *P* values were calculated using paired t-tests.

Having identified a miR-19a binding site in four well known regulators of the JAK-STAT pathway (SOCS1, SOCS3, SOCS5 and Cul5), we were interested in investigating the effect of miR-19a on these genes. The Huh-7 cell line expresses low endogenous levels of miR-19a (Figure **S4**), and therefore serves as an appropriate cell line to study miR-19a function, since miR-19a is readily detectable following transfection of its mimic (Figure 2A). Upon transfection of miR-19a, there was a significant down regulation of SOCS3 (p=0.0010), when compared to control cells. SOCS1 (p=0.2370), SOCS5 (p=0.1737) and Cul5 (p=0.4310) expression were not statistically significantly reduced (Figure 2B). SOCS2 mRNA, that does not contain a 3’UTR target sequence for miR-19a, did not decrease in the presence of miR-19a (p=0.1526), demonstrating specificity of miR-19a for target interaction. These results indicate that the expression of the JAK-STAT pathway inhibitor, SOCS3, is significantly regulated by miR-19a.

**Figure 3:**
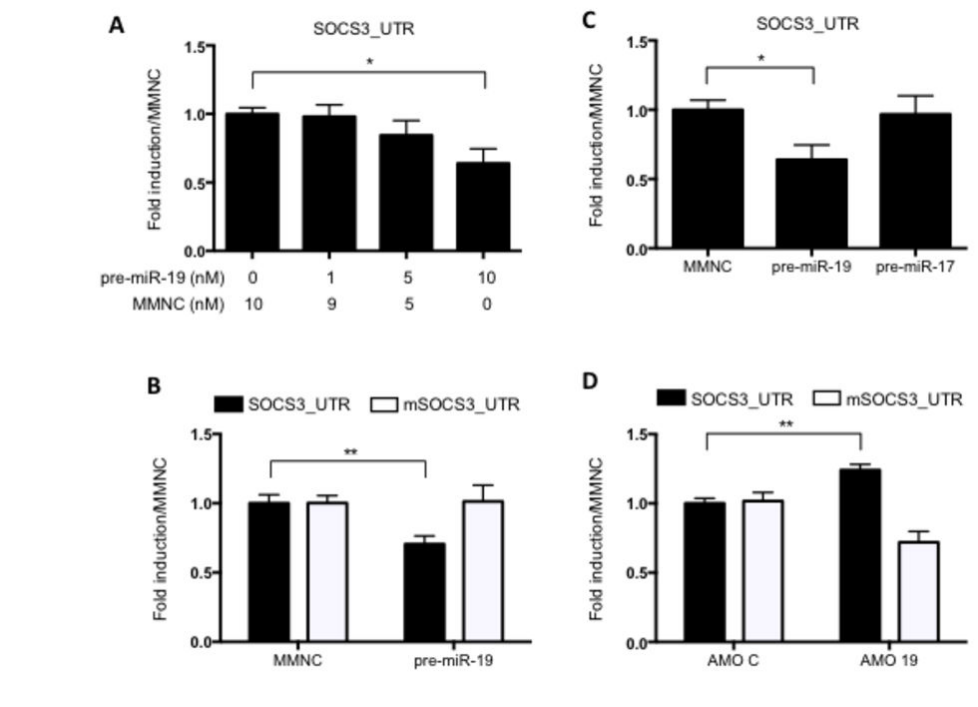
miR-19a destabilises SOCS3 3’UTR expression. Huh7 cells were transfected with (A) SOCS3 3’UTR *Renilla* reporter (SOCS3_UTR) and varying concentrations of double-stranded miR-19a mimic (pre-miR-19a) and MMNC control as indicated, (B) SOCS3_UTR or mutated SOCS3_UTR *Renilla* reporter with double-stranded (10 nM) miR-19a mimic (pre-miR-19a) or MMNC control, (C) SOCS3_UTR *Renilla* reporter and double-stranded (10 nM) miR-19a mimic, miR-17 mimic and MMNC control, (D) SOCS3_UTR or mutated SOCS3_UTR *Renilla* reporter with miR-19a antagomir (AMO 19) and control antagomir (AMO C). In each experiment, *Renilla* luciferase expression was normalised to firefly luciferase tranfection control and plotted as fold induction relative to MMNC or AMO control. (A, C) Data was averaged from biological triplicates and is representative of independent experiments. (C, D) Data was averaged from three independent experiments in biological triplicate. Two tailed *t*-tests are shown (*P < 0.05, **P < 0.01).

In order to confirm that SOCS3 is a target of miR-19a, a *Renilla* luciferase reporter containing the entire 3’UTR of human SOCS3 was generated (Figure **S2**). Upon transfection of increasing doses of miR-19a mimic, *Renilla* luciferase activity was significantly decreased (Figure 3A). Importantly, this effect did not occur when the miR-19a site within the SOCS3 3’UTR was mutated (Figure 3B). Overexpression of miR-17, a member of the miR-17-92 miRNA cluster to which miR-19a also belongs, did not recapitulate the effect of miR-19a on the SOCS3_3’UTR *Renilla* reporter, confirming target specificity of miR-19a for SOCS3 (Figure 3C). We also investigated if a miR-19a antagomir (AMO), which acts to bind and block any remaining endogenous miR-19a expressed in the cell, would have the opposite effect on SOCS3 3’UTR *Renilla* activity. Figure 3D illustrates that there is significantly enhanced *Renilla* luciferase activity of the wild-type SOCS3_UTR reporter in the presence of miR-19 antagomir. Interestingly, the miR-19a antagomir reduced *Renilla* luciferase activity of the mutated reporter in Huh7 cells, an effect we did not observe in HEK293T cells (data not shown), possibly demonstrating cell type specific variation. Together, these results confirm SOCS3 as a functional target of miR-19a, which may have implications for the regulation of the numerous pathways controlled by SOCS3.

### miR-19a reduces SOCS3 protein expression and enhances IFN-α and IL-6-induced pSTAT3

**Figure 4:**
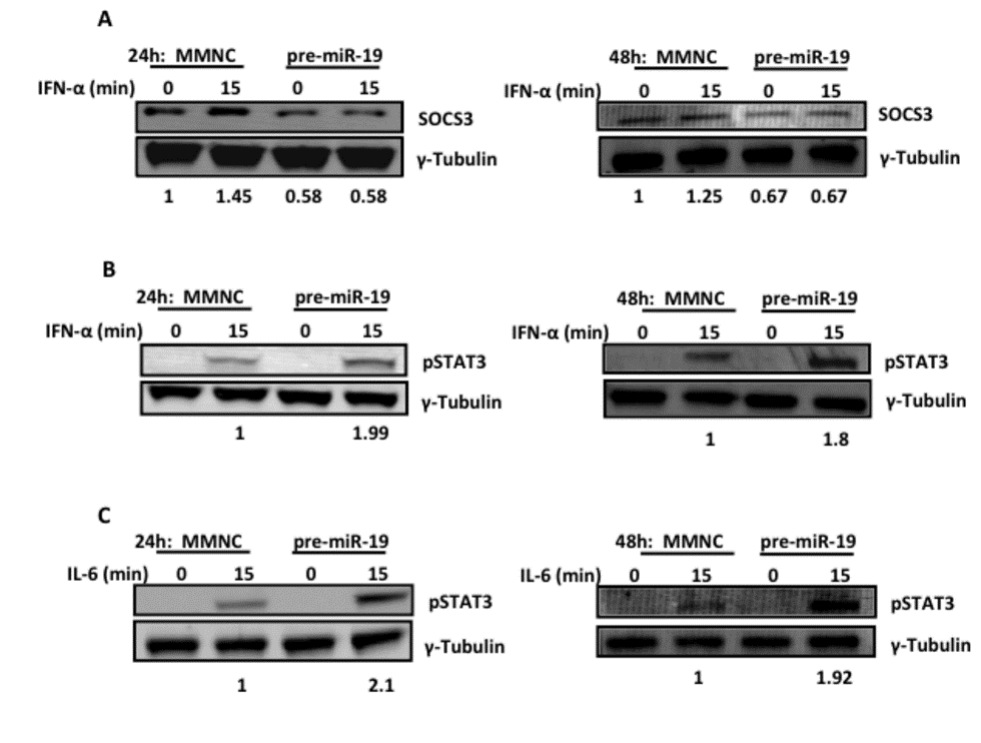
Decreased SOCS3 and increased pSTAT3 in presence of miR-19a. SOCS3 protein basal levels were reduced in Huh7s transfected with double-stranded miR-19a mimic (A), while pSTAT3 was increased following stimulation with IFN-α (B) and IL-6 (C) for 15min over 24 and 48h. Values below blots represent densitometric analysis of SOCS3 or pSTAT3 relative to γ-tubulin and normalised to MMNC=1(n=3).

Since miR-19a reduced SOCS3 mRNA and luciferase activity, we next examined its functional effect on SOCS3 protein and downstream signalling induced by IFN-α and IL-6, known to be regulated by SOCS3 [8,9]. Upon expression of miR-19a in Huh7 hepatocytes, SOCS3 protein levels decreased over 24 and 48h compared to MMNC (Figure 4A). Furthermore, the specific reduction in SOCS3 levels consistently enhanced IFN-α-induced pSTAT3 after 24, 48 or 72h expression of miR-19a, compared to MMNC expression (Figure **4B** **and** Figure **S5**). Since JAK-STAT signal transduction of IL-6 is also regulated by SOCS3 we analysed the effect of miR-19a-mediated SOCS3 reduction upon IL-6-induced pSTAT3. We found that, as with IFN-α, IL-6-mediated pSTAT3 levels were also enhanced in cells with miR-19a reduced SOCS3 expression, compared to MMNC treated cells (Figure 4C). Taken together these results indicate that miR-19a functionally regulates SOCS3 expression in order to promote STAT3-mediated signal transduction of IFN-α and IL-6.

## Discussion

Using KEGG database, InnateDB and TargetScan, we identified miR-19a sequence matches in the 3’UTRs of 12 JAK-STAT pathway proteins. We found that a miR-19a mimic significantly down regulated SOCS3 expression. Expression of SOCS1, SOCS5 and Cul5 mRNA was also reduced, albeit not significantly, indicating that miR-19a is not the primary regulator of these genes. In addition, the miR-19a mimic significantly reduced SOCS3 3’UTR reporter assays and SOCS3 protein expression in Huh7 hepatocytes. Furthermore, we found enhanced cytokine-induced STAT3 phosphorylation, possibly as a result of reduced SOCS3-mediated regulation of the IFN-α and IL-6 pathways. These results provide a novel functional role for miR-19a in the regulation of immune signalling and highlight it as a specific regulator of SOCS3.

The regulation of SOCS1 by miR-19 has been reported in mutiple myeloma cells [30]. We also observed a modest decrease in SOCS1 in the Huh7 hepatocyte cell line upon over-expression of double-stranded miR-19a mimic, however, there was a more significant impact on SOCS3. These differences could possibly be explained by variation between cell types. However, it is interesting to note that SOCS1 is also strongly targeted by miR-155, a miRNA induced by Toll-like receptors, RIG-I and viral infection [33]. It is plausible that there may be co-operation between miR-155 and miR-19 in the regulation of SOCS1, yet a separate and individual role for miR-19 regarding SOCS3. Having identified other miRNA binding sites in the 3’UTR of SOCS3 (Figure **S3B**), future investigations should aim to decipher the contribution of these miRNAs and/or any co-operation with miR-19a in the regulation of SOCS3.

SOCS3 negatively regulates the JAK-STAT pathway and is vital in controlling the duration and amplitude of JAK-STAT signalling. We found that miR-19a significantly targets SOCS3 and that its presence enhanced IFN-α and IL-6-induced pSTAT3, indicating that miR-19a may functionally contribute to the regulation of both anti-viral and pro-inflammatory responses. Recently, miR-19 and components of the miR-17-92 cluster have been shown to potentiate NF-κB activity by targeting negative regulators of nuclear factor (NF)-kB signaling, such as A20, RING finger protein (RNF) 11 and zinc finger and BTB domain containing (ZBTB) 16 [34], further highlighting a role for miR-19 and its contribution to pro-inflammatory responses.

miR-19a is one of six precursors (miR-17, miR-18a, miR-19a, miR-20a, miR-19b-1, and miR-92-1) transcribed as one common primary transcript from the miR-17-92 cluster located on chromosome 13 [35,36]. Several studies have linked the enhanced expression of the miR-17-92 cluster to cancer, including B-cell lymphoma and lung cancer [37,38]. In addition, more than 200 publications have established a role for this cluster in oncogenesis. Heightened activation of NF-κB and STAT3 are also significant promoters of cancer [39,40]. It is plausible that miR-19, which depletes negative regulators of both these pathways, acts to promote their activation and their contribution to cancer.

Boosting expression of the miR-17-92 cluster significantly diminishes replication of both HCV and Hepatitis B virus (HBV) in human hepatocytes and the liver, suggesting therapeutic potential for these regulators [40,41]. As administration of recombinant IFN-α is commonly used for the treatment of HCV and HBV, it is interesting that anti-viral IFN-α has been shown to induce miR-19 [42]. HCV induces SOCS3 expression, which is hypothesised to be partly responsible for the immunosuppression associated with chronic HCV infection [12]. Our finding of SOCS3 repression in response to miR-19a over-expression may provide additional explanation for the therapeutic value of IFN-α. It is clear that the expression of miR-19 and the miR-17-92 cluster is required to be kept in fine balance, where its expression is required to promote both anti-viral and pro-inflammatory responses, yet its overexpression may pre-dispose an individual to inflammatory disorders, cancer and viral infection.

In conclusion, we have found miR-19a to play a role in the regulation of SOCS3. Our results indicate that miR-19a can enhance JAK-STAT signalling, thus having the potential to amplify a potent mediating mechanism of several important biological processes. In fact, the regulatory effects of miR-19a may provide a missing link to our understanding of immune regulation and the progesssion of cancer and anti-viral immunity, that in the future may be harnessed to control inflammatory disorders associated with dysregulated JAK-STAT signalling.

## Supporting Information

Figure S1 Primer sequences used in Figure 2.
(PDF)

Figure S2 Human SOCS3 3’UTR sequence. The human SOCS3 3’UTR sequence was obtained from PubMed (Accession number NM_003955). The shaded grey sequence denotes primers designed to amplify the entire 3’UTR of SOCS3 from human genomic DNA, which was then cloned into the pRL-*Renilla* reporter plasmid. The seed sequence of miR-19a is highlighted in bold. Underlined sequence denotes the primers designed to perform site directed mutagenesis of the miR-19a seed region. The three bases that were mutated within the seed region are capitalized.
(PDF)

Figure S3 JAK/STAT pathway proteins identified by KEGG database and InnateDB and miRNA binding sites in the 3’UTRs of SOCS1, SOCS3 and SOCS5. (A) A combination of KEGG database (containing pathway maps representing molecular interaction and reaction networks) and InnateDB database (containing innate immunity interactions and pathways), identified 158 proteins in the JAK-STAT pathway. (B) The 3’UTR regions of SOCS1, SOCS3 and SOCS5 were screened for microRNA binding sites using Targetscan software.
(PDF)

Figure S4 Basal miR-19 relative expression in 293T and Huh7 cells. Total RNA was extracted from non-stimulated 293T and Huh7 cells. miR-19a was measured by qRT-PCR, where expression was normalised to U6 RNA and shown relative to 293T cells.
(PDF)

Figure S5 Decreased SOCS3 and increased pSTAT3 protein in the presence of miR-19a. Quantisation of (A) SOCS3 and (B) IFN-α-stimulated pSTAT3 over a time course of 24, 48 and 72h calculated using densitometry analysis of band intensity relative to γ-Tubulin and normalised to MMNC=1. Error bars are mean ± SD of three independent experiments at each time point.
(PDF)
