# Supplementary material for: miR-19a: An Effective Regulator of SOCS3 and Enhancer of JAK-STAT Signalling
Source: PLoS One. 2013 Jul 22;8(7):e69090. doi: 10.1371/journal.pone.0069090 (PMC3718810; doi:10.1371/journal.pone.0069090)
Supplement: Figure S1 — Primer sequences used in Figure 2. (PDF) [file pone.0069090.s001.pdf]

Primer Gene	Primer sequence
SOCS1 (upstream primer)	5'-TACTTGCCTGGAACCATGTG-3'
SOCS1 (downstream primer)	5'-AGCTGCTACAACAACCAGGG-3'
SOCS3 (upstream primer)	5'-ATCCTGGTGACATGCTCCTC-3'
SOCS3 (downstream primer)	5'-CAAATGTTGCTTCCCCCTTA-3'
SOCS5 (upstream primer)	5'-TTCGTGCATGTTTTTTGAACCA-3'
SOCS5 (downstream primer)	5'-GGAGCCCATCAATTCCATCA-3'
Cul5 (upstream primer)	5'-TCTGTTTTCGGATGTGCATGCAGT-3'
Cul5 (downstream primer)	5'-GCTCAGTACTCGTGCCTGTGCT-3'
SOCS2 (upstream primer)	5'-TCGGTCAGACAGGATGGTACTG-3'
SOCS2 (downstream primer)	5'-TTGGTCCAGCTGATGTTTTAACA-3'

Figure S1.
