# Supplementary material for: miR-19a: An Effective Regulator of SOCS3 and Enhancer of JAK-STAT Signalling
Source: PLoS One. 2013 Jul 22;8(7):e69090. doi: 10.1371/journal.pone.0069090 (PMC3718810; doi:10.1371/journal.pone.0069090)
Supplement: Figure S2 — The human SOCS3 3’UTR sequence was obtained from PubMed (Accession number NM_003955). The shaded grey sequence denotes primers designed to amplify the entire 3’UTR of SOCS3 from human genomic DNA, which was then cloned into the pRL-Renilla reporter plasmid. The seed sequence of miR-19a is highlighted in bold. Underlined sequence denotes the primers designed to perform site directed mutagenesis of the miR-19a seed region. The three bases that were mutated within the seed region are capitalized. (PDF) [file pone.0069090.s002.pdf]

gggtaaagggcgcaaagggcatgggtcgggagaggggacgcaggcccctctcctccg
tggcacatggcacaagcacaagaagccaaccaggagagagtcctgtagctctggggg
gaaagagggcggacaggcccctccctctgccctctccctgcagaatgtggcaggcgg
acctggaatgtgttggagggaaggggagtaccacctgagtctccagcttctccgga
ggagccagctgtcctggtgggacgatagcaaccacaagtggattctccttcaattcc
tcagcttcccctctgcctccaaacaggggacacttcgggaatgctgaactaatgaga
actgccagggaatcttcaaactttccaacggaacttgtttgctctttgatttggttt
aaacctgagctggttgtggagcctgggaaaggtggaagagagagaggtcctgagggc
cccagggctgcgggctggcgaaggaaatggtcacacccccgcccaccccaggcgag
gatcctggtgacatgctcctctccctggctccggggagaagggcttggggtgacctg
aagggaaccatcctggtaccccacatcctctcctccgggacagtcaccgaaaacaca
ggttccaaagtctacctggtgcctgagagcccagggcccttcctccgttttaagggg
gaagcaacatttggaggggatggatgggctggtcagctggtctccttttcctactca
tactataccttcctgtacctgggtggatggagcgggaggatggaggagacggacat
ctttcacctcaggctcctggtagagaagacaggggattctactctgtgcctcctgac
tatgtctggctaagagattcgccttaaatgctccctgtcccatggagagggacccag
cataggaaagccacatactcagcctggatgggtggagaggctgagggactcactgga
gggcaccaagccagcccacagccagggaagtggggagggggggcggaaacccatgcc
tcccagctgagcactgggaatgtcagcccagtaagtattggccagtcaggcgcctcg
tggtcagagcagagccaccaggtcccactgccccgagccctgcacagccctccctcc
tgcctgggtgggggaggctggaggtcattggagaggctggactgctgccaccccggg
tgctcccgctctgccatagcactgatcagtgacaatttacaggaatgtagcagcgat
ggaattacctggaacagtttttgtttttgtttttgtttttgtttttgtggggggg
gcaactaaacaaacacaaagtattctgtgtcaggtattgggctggacagggcagttg
tgtgttggggtggtttttttctctatttttttgtttgtttcttgtttttaataatg
tttacaatctgcctcaatcactctgtcttttataaagattccacctccagtcctctc
tcctccccccctactcaggcccttgaggctattaggagatgcttgaagaactcaacaa
aatcccaatccaagtcaaac**tttGCAca**tatttatatttatattcagaaaagaaaca
tttcagtaattta

Figure S2
