# Supplementary material for: miR-19a: An Effective Regulator of SOCS3 and Enhancer of JAK-STAT Signalling
Source: PLoS One. 2013 Jul 22;8(7):e69090. doi: 10.1371/journal.pone.0069090 (PMC3718810; doi:10.1371/journal.pone.0069090)
Supplement: Figure S3 — (A) A combination of KEGG database (containing pathway maps representing molecular interaction and reaction networks) and InnateDB database (containing innate immunity interactions and pathways), identified 158 proteins in the JAK-STAT pathway. (B) The 3’UTR regions of SOCS1, SOCS3 and SOCS5 were screened for microRNA binding sites using Targetscan software. (PDF) [file pone.0069090.s003.pdf]

A

Genes							
TPO	IFNK,	IL6	IL12RB1	IFNGR1	EP300	PIAS3	IFNB1
EPO	IFNE,	IL12A	IL12RB2	IFNGR2,	SOCS1	PIAS4	IFNW1
CSH1,	IL10	IL11	IL23R	IL10RA	SOCS2	PIAS1	IL13
GH1,	IL24	OSM	CSF2RA,	IL10RB	SOCS3,	PIAS2	TSLP
GH2	IL19	LIF	IL5RA	IL20RA	SOCS4,	CBLC	CSF3R
PRL	IL20	CNTF	IL2RA	IL20RB	SOCS5	CBL	LEPR
IFNG,	IL22	CLCF1	IL2RB	IL22RA1	SOCS7,	CBLB	IFNAR1
IFNA1,	IL26	CTF1	IL2RG	IL22RA2	CISH	PTPN11	IFNAR2
IFNA2,	IL28A	CSF3	IL4R,	IL28RA	PIM1	GRB2	IRF9
IFNA4,	IL28B	LEP	IL7R,	JAK1	MYC	SOS1,	CREBBP
IFNA5	IL29	IL12B	IL9R	JAK2	CCND1	SOS2	STAM2
IFNA6	IL3	IL23A	IL15RA	JAK3	CCND2	PIK3CA	STAM
IFNA7	CSF2	IL3RA,	IL21R	TYK2	CCND3	PIK3CB	AKT2
IFNA8	IL5	CSF2RB	IL13RA1	STAT1	BCL2L1	PIK3CD	PTPN6
IFNA10	IL2	IL6R	IL13RA2	STAT2	SPRED1	PIK3CG	PIAS1
IFNA13	IL4	IL11RA	CRLF2	STAT3	SPRED2	PIK3R5	CSH2
IFNA14	IL7,	OSMR	EPOR	STAT4	SPRY3,	PIK3R1	SPRED3
IFNA16	IL9,	LIFR	GHR	STAT5A	SPRY1	PIK3R2	AKT3
IFNA17	IL15	CNTFR	PRLR	STAT5B,	SPRY2	PIK3R3,	
IFNA21	IL21	IL6ST,	MPL	STAT6	SPRY4	AKT1	

B

SOCS1	SOCS3	SOCS5
miR-155	miR-218	miR-218
miR-221/222	miR-455-5p	miR-33a-3p
miR-30abcdef	miR-221/222	miR-101
**miR-19ab**	miR-30abcdef	miR-124
	**miR-19ab**	miR-9
	miR-148ab/152	miR-138
	miR-383	**miR-19ab**
	miR-18abcd	miR-130ac
	miR-203	

Figure S3
