# Supplementary figures and images for: miR-19a: An Effective Regulator of SOCS3 and Enhancer of JAK-STAT Signalling
Source: PLoS One. 2013 Jul 22;8(7):e69090. doi: 10.1371/journal.pone.0069090 (PMC3718810; doi:10.1371/journal.pone.0069090)

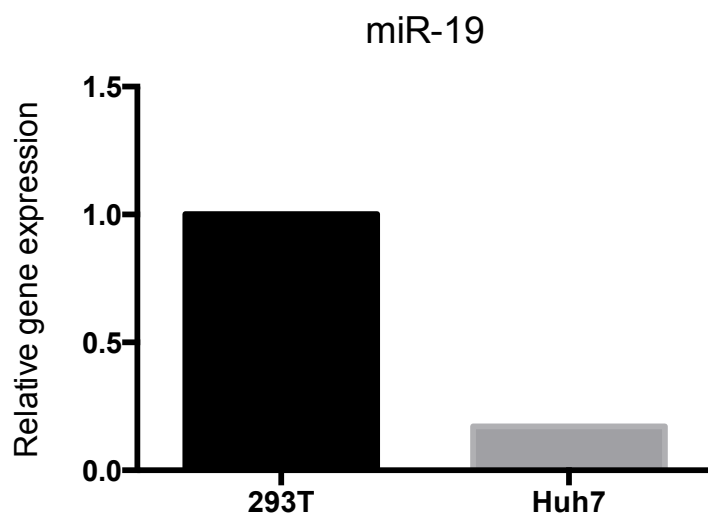

Figure S4

Supplement: Figure S4 — Total RNA was extracted from non-stimulated 293T and Huh7 cells. miR-19a was measured by qRT-PCR, where expression was normalised to U6 RNA and shown relative to 293T cells. (PDF) [file pone.0069090.s004.pdf]

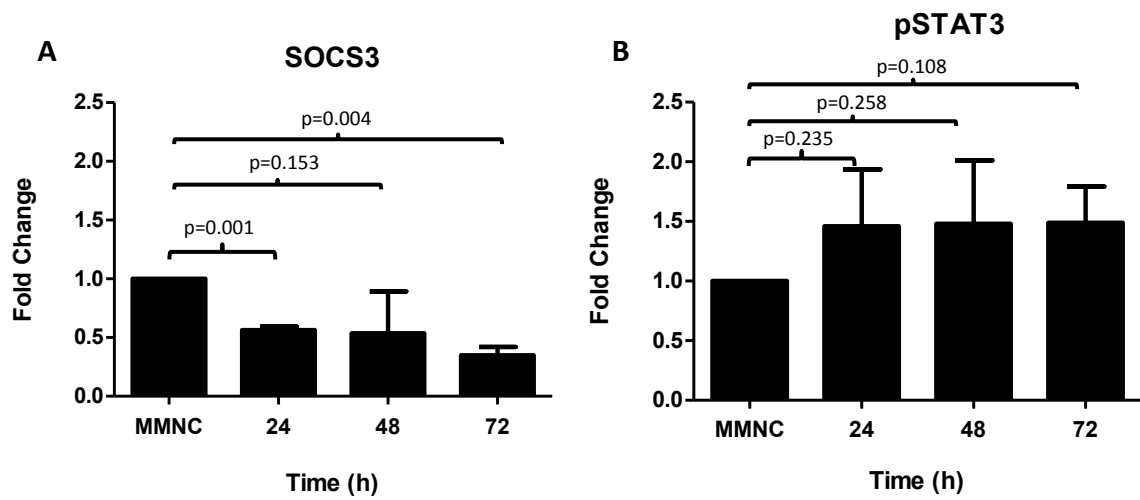

Figure S5

Supplement: Figure S5 — Quantisation of (A) SOCS3 and (B) IFN-α-stimulated pSTAT3 over a time course of 24, 48 and 72h calculated using densitometry analysis of band intensity relative to γ-Tubulin and normalised to MMNC=1. Error bars are mean ± SD of three independent experiments at each time point. (PDF) [file pone.0069090.s005.pdf]
